# Supplementary figures and images for: No evidence for genome editing in mouse zygotes and HEK293T human cell line using the DNA-guided Natronobacterium gregoryi Argonaute (NgAgo)
Source: PLoS One. 2017 Jun 13;12(6):e0178768. doi: 10.1371/journal.pone.0178768 (PMC5469460; doi:10.1371/journal.pone.0178768)

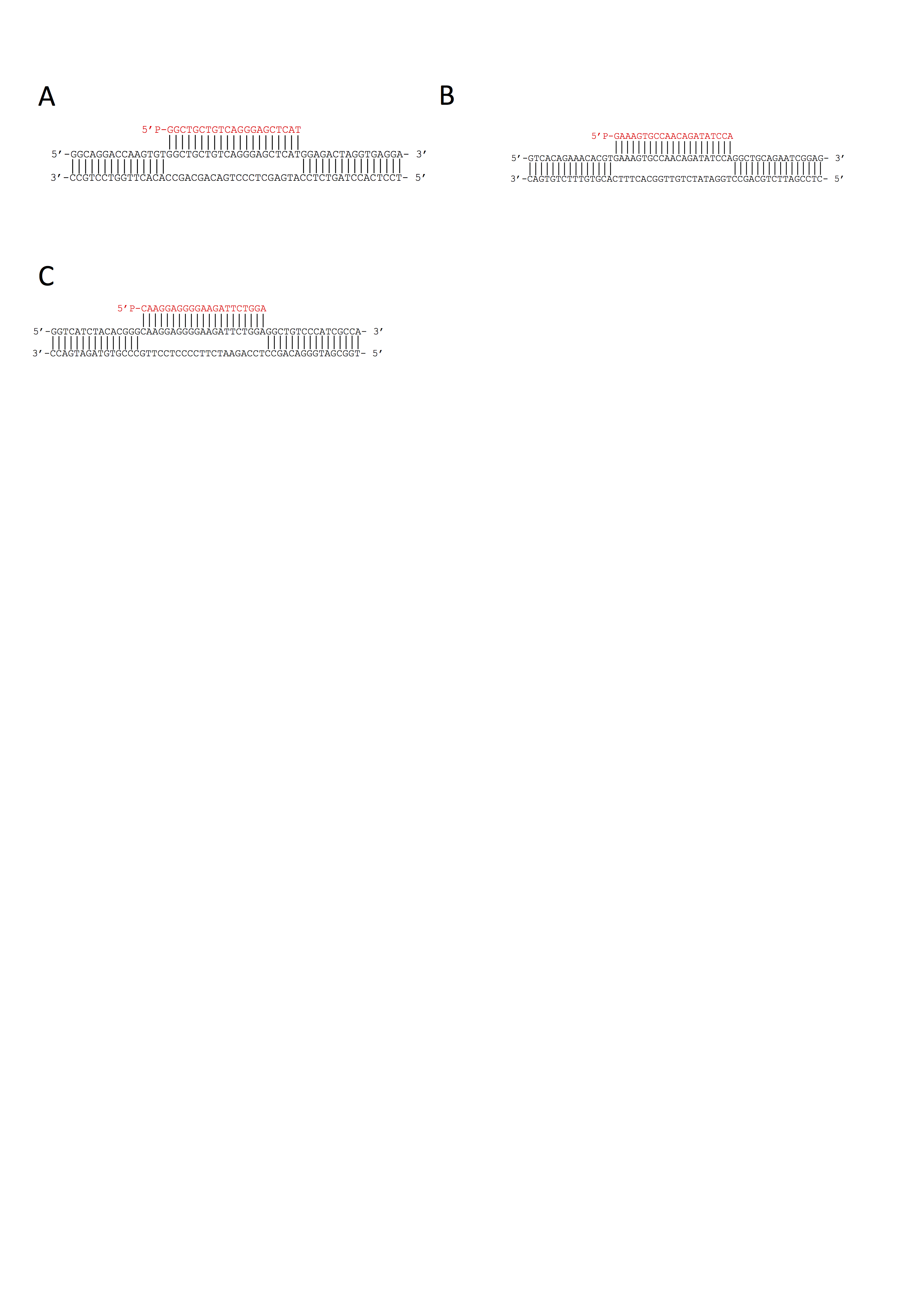

Supplement: S1 Fig — (A) DNA sequence indicating the locus targeted for the exon 5 from Tet-1. B) Exon 4 from Tet-2 C) Exon 5 from Tet-3. The gDNA sequence is indicated in red. (TIFF) [file pone.0178768.s001.tiff]

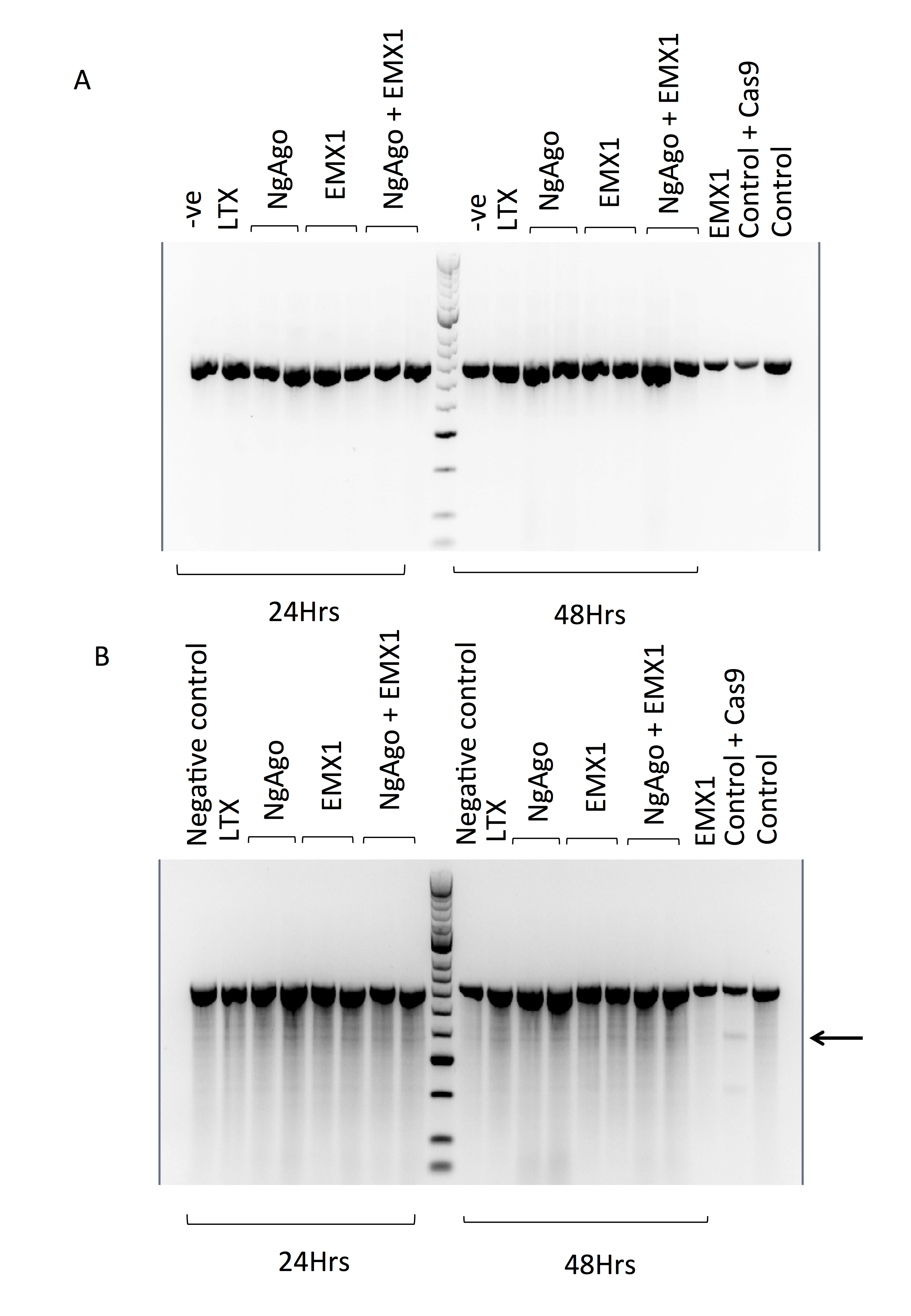

Supplement: S2 Fig — Gel electrophoresis (2%) of the PCR for EMX1 in HEK293T cells at 24 and 48 hours post-lipofection. The control samples were: The endogenous DNA (negative control), EMX1 DNA and lipofection reagent (LTX). The HEK293T cells were transfected with NgAgo alone, EMX1 gDNA alone or co-transfected with NgAgo and EMX1 gDNA. A control DNA was successfully edited with CRISPR/Cas9 (+ Cas9 control) and without the addition of Cas9 (- Cas9 control). (A) Represents the PCR product for EMX1 (B) T7E1 assay. The arrows indicate the formation of heteroduplexes using CRISPR/Cas9 genome editing system. (TIFF) [file pone.0178768.s002.tiff]

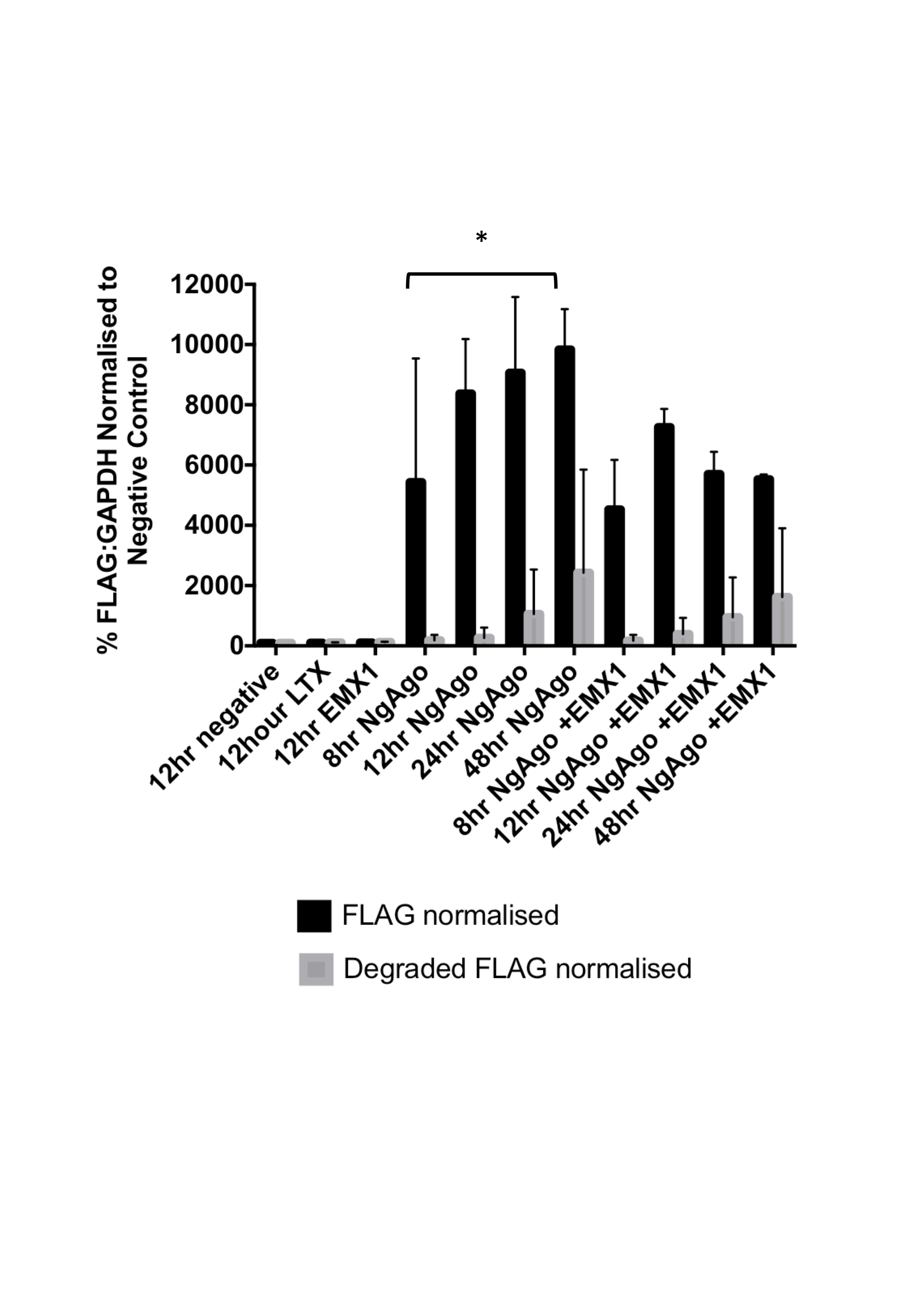

Supplement: S3 Fig — NgAgo was transfected or co-transfected in HEK293T cells and the protein expression level for NgAgo was monitored over 48 hours by Western Blot using a monoclonal Flag tag antibody. The dark bars represent the normalized NgAgo expression level to GAPDH housekeeping control whereas the grey bars indicate the degradation of NgAgo protein normalized to GAPDH level. The experiment was performed on duplicate from 2 biological samples. * Represents p < 0.05. (TIFF) [file pone.0178768.s003.tiff]
